# Naringin Ameliorates Skeletal Muscle Atrophy and Improves Insulin Resistance in High-Fat-Diet-Induced Insulin Resistance in Obese Rats

**DOI:** 10.3390/nu14194120

**Published:** 2022-10-04

**Authors:** Chutimon Termkwancharoen, Wachirawadee Malakul, Amnat Phetrungnapha, Sakara Tunsophon

**Affiliations:** 1 Department of Physiology, Faculty of Medical Science, Naresuan University, Phitsanulok 65000, Thailand; 2 Department of Biochemistry, Faculty of Medical Science, Naresuan University, Phitsanulok 65000, Thailand; 3 Center of Excellence for Innovation in Chemistry, Naresuan University, Phitsanulok 65000, Thailand

**Keywords:** high-fat diet, obesity, naringin, insulin resistance, skeletal muscle, atrophy

## Abstract

Obesity causes progressive lipid accumulation and insulin resistance within muscle cells and affects skeletal muscle fibres and muscle mass that demonstrates atrophy and dysfunction. This study investigated the effects of naringin on the metabolic processes of skeletal muscle in obese rats. Male Sprague Dawley rats were divided into five groups: the control group with normal diet and the obese groups, which were induced with a high-fat diet (HFD) for the first 4 weeks and then treated with 40 mg/kg of simvastatin and 50 and 100 mg/kg of naringin from week 4 to 8. The naringin-treated group showed reduced body weight, biochemical parameters, and the mRNA expressions of protein degradation. Moreover, increased levels of antioxidant enzymes, glycogen, glucose uptake, the expression of the insulin receptor substrate 1 (IRS-1), the glucose transporter type 4 (GLUT4), and the mRNA expressions of protein synthesis led to improved muscle mass in the naringin-treated groups. The in vitro part showed the inhibitory effects of naringin on digestive enzymes related to lipid and glucose homeostasis. This study demonstrates the potential benefits of naringin as a supplement for treating muscle abnormalities in obese rats by modulating the antioxidative status, regulating protein metabolism, and improved insulin resistance in skeletal muscle of HFD-induced insulin resistance in obese rats.

## 1. Introduction

Obesity is a major public health problem worldwide; it has been officially recognized as a chronic disease that involves the excess intake of a high-fat diet (HFD), which affects lipid accumulation in the skeletal muscles, leading to the dysfunction of muscle [1]. A previous study stated that obesity-related changes caused an imbalance between both muscle protein synthesis and degradation due to the suppression of the IRS-1/AKT/mTOR signalling pathway. In addition, obesity shows alterations in the characteristics of the muscle fibres by a decrease in size, and a reduction in fibre numbers creates a decline in muscle strength caused by the loss of skeletal muscle mass or atrophy [2].

A HFD profoundly affects several physiological and pathological mechanisms and is a major risk factor for metabolic syndromes such as hypertension, dyslipidaemia, and insulin resistance to diabetes mellitus, and has the ability to reduce sensitivity to insulin [3]. There is a positive association between high fat diet consumption and obesity. Excessive HF consumption can induce obesity, resulting in the overproduction of reactive oxygen species (ROS) and oxidative stress from high-fatty-acid β-oxidation. This can later stimulate the secretion of pro-inflammatory cytokines, which subsequently leads to the dysregulation of metabolic homeostasis and aggravation of other health problems [4,5]. Previous studies showed that obesity related to insulin resistance was due to fat accumulation that causes increased triglycerides (TG) and free fatty acids (FFAs) accumulation in skeletal muscles, which affects the increase in oxidative stress and a decrease in antioxidants. This leads to the enhancement of ROS production [1,6], which interferes with the insulin signalling pathway and induces a decrease in muscle performance and regeneration. Therefore, excess FFAs and ROS products are important factors that reduce insulin response due to suppressed insulin intracellular signalling pathways that induce insulin resistance within the muscle cells. Under normal conditions, insulin plays an important role by regulating the metabolism of the skeletal muscles, which promotes glucose transport, glycogen synthesis, and protein metabolism via the IRS-1/AKT/mTOR signalling pathway. Insulin resistance may be caused by an abnormality in the signal transduction to activate the insulin receptors (IRS-1) and can impair both the glucose transporter type 4 (GLUT4) to the cell membrane and glycogen synthesis within the skeletal muscles, which affects the increase in both insulin and glucose in plasma. Insulin resistance also causes reduced glycogen content in muscles. This leads to reduced protein synthesis and energy production in muscle, which has a direct effect on muscle loss. 

Currently, statins are the most widely prescribed lipid-lowering agents that inhibit hydroxymethyl glutaryl-coenzyme A reductase (HMG-CoA reductase), which is related to endogenous cholesterol synthesis [7]. Statins, which include simvastatin, can cause serious side effects in skeletal muscle tissue, such as pain, myopathy, and rhabdomyolysis [8]. From several studies, they showed that simvastatin (SIM) caused myotoxicity and muscle atrophy [7,8,9], while another study found no evidence of side effects of statin on the morphology of muscle at the molecular, cellular, or whole tissue levels in healthy individuals [10]. A recent study suggested that SIM induces insulin resistance and is associated with glucose metabolism impairment and new-onset diabetes. Therefore, we were interested in finding an alternative remedy that incorporated natural polyphenolic compounds found in certain fruits, and compared its effectiveness with simvastatin which is one among the most frequently used for dyslipidaemic treatment. Naringin contains various biological properties that include anti-oxidants [11] and have few or no side effects. Previous studies have shown that naringin is a major flavonoid found in citrus fruits, such as pomelo and grapefruits [12,13], and its supplementation decreased plasma lipids, total cholesterol, and triglycerides in an experimental model of high-cholesterol-diet-fed rats [14]. Naringin was also responsible for increasing the amount of GLUT4 and glucose uptake in the L6 myotube cells [15]. In this study, we investigated the mechanism of naringin that affects the metabolic processes and enhanced insulin sensitivity of the skeletal muscles in the HFD-induce insulin resistance obese rat model. Our results demonstrated the robust evidence for the benefits of naringin found in grapefruits. Therefore, instead of using medicines that causes side-effects in the muscle tissues, naringin can be used as an alternative for treating the dysfunction of skeletal muscles related to obesity.

## 2. Materials and Methods

### 2.1. Preparation of Samples

Simvastatin (Sigma-Aldrich, St. Louis, MO, USA) and the naringin powder were purchased from the Sigma-Aldrich Company (Cat. No. 10236-47-2, St. Louis, MO, USA).

### 2.2. In Vitro

The total phenolic content (TPC) and the antioxidant capacity were measured using the 2,2-diphenyl-1-picrylhydrazyl (DPPH) assay, and the 2,2′-azino-bis (3′-ethylbenzthiazoline-6-sulphonic acid) (ABTS). Free radical scavenging activity of naringin was measured using a slightly modified method as described previously by Janson [16]. In addition, the effects of naringin on the α-glucosidase, the cholesterol esterase, and the pancreatic lipase inhibition assays were measured according to a previously described method [17,18,19].

#### 2.2.1. The α-Glucosidase Inhibition Assay

The effect of naringin on the α-glucosidase inhibition assay was measured according to a previously described method [17]. Briefly, the α-glucosidase enzyme was dissolved in a phosphate buffer saline. This was followed by adding the mixtures of α-glucosidase and the samples, and then incubated at 37 °C for 5 min; then, p-nitrophenyl-a-D-glucopyranoside (p-NPG) was added to initiate a reaction. This mixture was then incubated at 37 °C for 20 min, and the reaction was stopped by using Na_2_CO_3_. The α-glucosidase activity was measured at an absorbance of 405 nm, using a microplate reader. The acarbose was used as a positive control. 

#### 2.2.2. The Cholesterol Esterase Inhibition

The study of the effect of naringin on the cholesterol esterase inhibition assay was measured according to a previously described method [18]. The cholesterol esterase enzyme was dissolved in a phosphate buffer saline. This was followed by adding the mixtures of cholesterol esterase enzyme and the samples, incubation with taurocholic acid and p-NPB in the PBS solution, then incubation for 5 min at room temperature. The cholesterol esterase activity was measured at an absorbance of 405 nm by using a microplate reader. Simvastatin was used as a positive control. 

#### 2.2.3. The Pancreatic Lipase Inhibition Assay

The effect of naringin on the pancreatic lipase inhibition assay was measured according to a previously described method [19]. Briefly, the mixture contained samples, and pancreatic lipase enzyme and Tris-HCl pH 8.2 were mixed. The reaction was initiated by adding 4-nitrophenyl laurate followed by incubation at 37 °C for 2 h. The pancreatic lipase activity was measured the absorbance at 400 nm by microplate reader. Orlistat was used as a positive control.

### 2.3. Animal Groups and Treatments

Thirty-five male Sprague-Dawley rats (4 wk. old) weighing between 180 and 200 g were purchased from the Nomura Siam International, Bangkok, Thailand. All the rats were kept in the animal houses at the Centre for Animal Research, Naresuan University, Phitsanulok, Thailand, under controlled temperature at 22 ± 1 °C and relative humidity of 55 ± 10% in a 12 h light and dark cycle. They were acclimatized for 1 week, fed a commercial rodent diet, and allowed water ad libitum. 

The rats were randomly divided into five groups: the control rats (control), the high-fat diet (HFD) group, the HFD plus the 40 mg/kg BW of simvastatin (positive control; HFD + SIM) group, the HFD plus naringin at a dose of 50 mg/kg BW (HFD + N50) group, and the HFD plus naringin at a dose of 100 mg/kg BW (HFD + N100) group. All the rats were given water and food daily, and their body weight was measured every week throughout the experiment for 8 weeks. The simvastatin- and naringin-treated groups were orally gavaged with simvastatin at 40 mg/kg BW and naringin at 50 and 100 mg/kg BW, respectively, while an equal volume of distilled water was given to the control and HFD groups by gavage from week 4 to week 8. After 8 weeks of experiment, the rats were anesthetized by intraperitoneal injection with 50 mg/kg body weight of thiopental sodium. Their blood was then collected, and the quadriceps muscle were removed, weighed, and stored at −80 °C for subsequent experiments. The HFD contained 36% of total energy intake from fats (4.5 g of fat, 14 g of milk fat, and 1.5 g of cholesterol powder), while the normal food contained 10% of total energy fat (4.5 g of fat).

### 2.4. Oral Glucose Tolerance Test

After the rats were fasted for 12 h, they received 2 g/kg body weight of glucose solution by oral gavage. Blood samples from all the rats were obtained from the vein in their tails and then measured by a glucometer (ACCU-CHEK performa, Germany) at 0, 30, 60, 90, and 120 min to determine the level of glucose in each rat. The result of the blood glucose was shown as the area under the curve (AUC) [16].

### 2.5. Biochemical Analyses

The blood glucose was measured using a glucometer after fasting the rats for 12 h at the beginning and the end of the experiment. The levels of insulin in the serum were measured using a Rat/Mouse Insulin ELISA Kit (EDM Millipore, Burlington, MA, USA), according to the manufacturer’s instructions. Homeostasis model assessment of insulin resistance (HOMA-IR) was calculated using the formula: HOMA-IR = (Fasting blood glucose × Fasting serum insulin)/22.5 [16]. The total cholesterol (TC), triglyceride (TG) assay kits (Human Gesellschaf fur biochemical und diagnostica mbH, Wiesbaden, Germany), and creatine kinase (CK) and the lactate dehydrogenase (LDH) assay kits (Sigma-Aldrich, St. Louis, MO, USA) were all used according to the manufacturer’s instructions. The aspartate aminotransferase (AST) and alanine aminotransferase (ALT) in the serum were analysed by Medilab (Phitsanulok, Thailand).

### 2.6. Muscle Homogenization

The quadriceps muscle tissues were homogenized in PBS, centrifuged at 1000× *g* for 20 min at 4 °C, and the supernatants were assessed for the antioxidant activities. 

### 2.7. The Level of Superoxide Dismutase

The SOD levels in the samples being measured at a wavelength of 420 nm after adding Tris buffer (50 mmol/L Tris/1 mM EDTA) pH 8.2 and 4.5 mmol/L Pyrogallol in the cuvette that modified from Chung [20] and Prasomthong [21]. 

### 2.8. The Level of Catalase

The CAT levels in samples were measured at a wavelength of 240 nm after adding 50 mM PBS pH 7.0 and 30% hydrogen peroxide (H_2_O_2_) in the cuvette, followed by Hadwan [22].

### 2.9. The Level of Lipid Peroxidation

The level of lipid peroxidation was measured according to the method of Zhang [23]. The samples were mixed with solution containing 0.37% (*w*/*w*) of Thiobarbituric Acid (TBA), 0.25 N Hydrochloric Acid (HCl), and 15% *w*/*w* Trichloroacetic Acid (TCA) (Poch, Sowinskiego, Poland), incubated at 95 °C for 15 min, and then centrifuged at 4800× *g* at room temperature for 10 min. The product of the lipid peroxidation was the Malondialdehyde (MDA), with its absorbance measured at 532 nm, which was calculated based on a standard curve using 1,1,3,3-Tetramethoxypropane (TMP) as a standard. 

### 2.10. Measurement of Glucose Uptake in the Muscle Tissues

We followed the method previously reported by Janson [16] and Hassan et al. [24] and collected 90–150 mg of each of the quadriceps tissues, then soaked them in the Kreb’s- Ringer bicarbonate (KRB) buffer in the presence of 95% O_2_ and 5% CO_2_ at 37 °C. The muscles were then aerated in KRB solution containing insulin and glucose and a solution absence insulin, then incubated at 37 °C. The glucose concentrations were analysed using the Glucose (GO) assay kit (Sigma-Aldrich, St. Louis, MO, USA) by measuring the KRB solution containing a known concentration of glucose in the presence and absence of insulin. The solution was determined before and after the incubation period for 30 min. After that, the glucose concentration in the solution before and after the experiment was calculated to compare the muscle glucose uptake between treatment groups.

### 2.11. Measurement of Glycogen in the Skeletal Muscle

The skeletal muscle glycogen levels were determined as previously described by Nader [25]. A 200 mg sample of the quadriceps was collected in potassium hydroxide (KOH), then 2 mL of ethanol was added. Samples were incubated then centrifuged for 20 min at 24,000× *g*. The pellets were dissolved in 1 mL of distilled water. Then, the mixture of phenol and sulfuric acid was added and incubated on ice for 30 min. The samples were measured using a GO assay kit (Sigma-Aldrich, St. Louis, MO, USA) at a wavelength of 540 nm. 

### 2.12. RT-PCR Analysis

Total RNA was extracted from the quadriceps tissues using Ribozol reagent (VWR Life Science AMRESCO, Radnor, PA, USA). The cDNA was obtained by reverse transcription from total RNA. The PCR was carried out using a PCR thermocycling machine (Thermo Fisher Scientific, Waltham, MA, USA) that followed the primer sequences and annealing temperatures shown in Table 1. The purified PCR product and the loading dye were mixed together, then loaded into the gel and observed using a gel document machine (ImageQuant LAS 500, GE Healthcare Bio-Sciences AB, Uppsala, Sweden). This was followed by a band of RNA expression being analysed using the Image J program. The gene expression levels, including superoxide dismutase 1 (*SOD1*), catalase (*CAT*), muscle RING-finger protein-1 (*MuRF-1*), muscle atrophy F-box (*Atrogin-1*), mammalian target of rapamycin (*mTOR*), and peroxisome proliferator-activated receptor co-activator-1α (*PGC-1α*), were analysed, and *β-actin* was used as an internal control.

### 2.13. Western Blotting Analysis

The quadriceps muscle was homogenized in RIPA Lysis Buffer with protease inhibitor cocktail and centrifuged at 20,000× *g* for 15 min at 4 °C. Then, the supernatants were determined by the BCA Protein Assay Kit. An equal number of proteins (80 μg) were loaded in SDS-PAGE gel and transferred to a polyvinylidene fluoride (PVDF) membrane. The membrane was incubated with the primary antibodies of GLUT4 (1:500, cat. no. AF5386, Affinity bioscience, Cincinnati, OH, USA), IRS-1 (1:1000, cat. no. E-AB-669799, Elabscience Biotechnology Inc., Houston, TX, USA), and β-actin (1:2000, cat. no. E-AB-20031, Elabscience Biotechnology Inc., Houston, TX, USA) at 4 °C overnight, after being incubated in 5% skim milk for 1 h at room temperature. The membranes were washed three times for 5 min with TBST buffer and then incubated with the secondary antibodies for 1 h at room temperature. The proteins from the samples were then observed using a chemiluminescence machine (ImageQuant LAS 500, GE Healthcare Bio-Sciences AB, Uppsala, Sweden). This was followed by the band intensities of the proteins being analysed using the Image Lab program.

### 2.14. Hematoxylin and Eosin (H&E) Staining

We collected the whole quadriceps of each leg after the rats were euthanized and then used lateral part of the quadriceps, as previously described by Tosolini [26]. In addition, each sample was labelled with a coding number, and the muscle histology of each rat was determined for three slides with five random areas per slide. The quadricep muscles were fixed in 10% neutral buffered formalin, cut into 5 µm thick sections, and then stained with H&E. The cross-section areas (CSA), and diameter (minimal Feret’s diameter) of muscle fibres per unit area were calculated in five random fields of each section, as previously described [20,27]. Images of the stained sections were obtained under a light microscope and measured using Image J software.

### 2.15. Statistical Analysis

All data were analysed with One-Way Analyses of Variance (ANOVA) followed by Tukey’s multiple comparison test using GraphPad Prism, version 5 (GraphPad Software Inc., San Diego, CA, USA). The statistical differences between the HFD and treatment groups were analysed with the paired Student’s t-test to determine the markers of injured muscles. Statistical significance was considered at *p* < 0.05, and the results are expressed as mean ± SEM.

## 3. Results

### 3.1. Effect of Naringin on In Vitro Study

The naringin compound showed that the total phenolic content, the DPPH, and the ABTS values were 1.72 mgGAE/gDW, 3.30 molAAE/gDW, and 0.17 molTE/gDW, respectively. Therefore, these results indicated that naringin had a rich phenolic content, which is one of the natural antioxidant compounds (Table 2).

An in vitro study on the digestive enzyme’s inhibitory activity of the naringin showed a strong inhibitory effect in a dose-dependent manner. The highest concentration of naringin showed maximum inhibition of α-glucosidase, cholesterol esterase, and pancreatic lipase were 81.22 ± 0.35, 97.26 ± 0.88, and 71.21 ± 3.29 %, respectively (Table 2). 

### 3.2. Effect of Naringin on the Body Weight of High-Fat-Diet-Fed Rats

The mean daily food intake was significantly increased in the HFD group, which led to a body weight gain of 24.08% and showed that the value of the adiposity index was 6.39 ± 0.58%, which was higher than the control group (*p* < 0.01). In addition, Lee’s index and the Body Mass Index (BMI), are a means of determining obesity in rats (Lee’s index > 310 g/cm, obesity). Therefore, Lee’s index and the BMI of the HFD group were significantly higher than the control group (*p* < 0.001), whereas the naringin group treated with 100 mg/kg and the simvastatin group had significantly reduced final body weight (*p* < 0.05, and *p* < 0.01, respectively), adiposity index (%) (*p* < 0.001), BMI (*p* < 0.01, and *p* < 0.05, respectively), and Lee’s index (*p* < 0.05), while Lee’s index in the simvastatin group showed no statistical differences when compared with the HFD group (Table 3). The calorie intake of the HFD group was significantly higher than the control group (*p* < 0.01), whereas the other groups showed no significant differences (Table 3).

### 3.3. The Effect of Naringin on Lipid Accumulation in the Muscle of High-Fat-Diet Rats

Levels of TC and TG in the muscle tissues of the HFD group had significantly increased when compared with the control group (*p* < 0.05 and *p* < 0.001, respectively). Conversely, the skeletal muscle tissues of the naringin- and simvastatin-treated groups had significantly decreased levels of TC (*p* < 0.001 and *p* < 0.01, respectively) and TG (*p* < 0.001), when compared with the HFD group (Table 3).

### 3.4. Effect of Naringin on Markers of Muscle Injury in Serum of High-Fat-Diet-Fed Rats

Muscle injury was measured by determining the CK, LDH, AST, and ALT in the serum. A significant increase in the levels of both AST and ALT activities in the HFD group were observed when compared with the control group (*p* < 0.001 and *p* < 0.01, respectively). The simvastatin group had significantly increased CK, AST, and ALT activities (*p* < 0.05, *p* < 0.001, and *p* < 0.01, respectively), while showing no statistical difference in the level of LDH, when compared with the control group. Interestingly, the rats treated with naringin had significantly decreased levels of CK in the serum when compared with the HFD + SIM group, and the LDH, and AST activities also showed significantly lower levels when compared with the HFD group (*p* < 0.05) (Table 3).

### 3.5. Effect of Naringin on Blood Glucose and Insulin Resistance in High-Fat-Diet-Fed Rats

**Figure 1:**
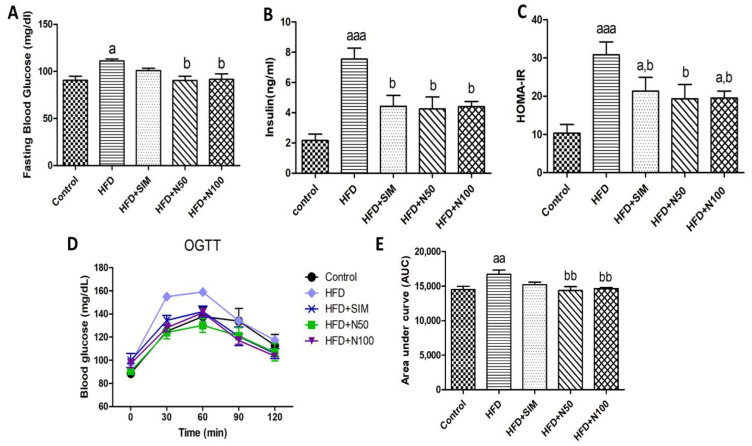
The effect of naringin on blood glucose and insulin levels in high-fat-diet-fed rats. FBG (**A**), insulin (**B**), HOMA-IR (**C**), OGTT (**D**), and AUC (**E**). Results are shown as mean ± SEM for 4 to 6 rats. * *p* < 0.05 when compared to week 0. ^a^ *p* < 0.05, ^aa^ *p* < 0.01, and ^aaa^ *p* < 0.001 vs. the control group; ^b^ *p* < 0.05, and ^bb^ *p* < 0.01 vs. the HFD group.

After 8 weeks of treatment, the fasted blood glucose (FBG) of the HFD group was significantly increased (*p* < 0.05, Figure 1A), which led to an increase in both insulin levels (Figure 1B) and HOMA-IR (Figure 1C), when compared with the control group (*p* < 0.001). The results demonstrated that insulin resistance was induced by the high-fat food fed to the rats. Conversely, the simvastatin- and naringin-treated groups had significantly reduced serum insulin, HOMA-IR, and the FBG, whereas the FBG of the HFD + SIM group showed no statistical difference when compared with the HFD group (*p* < 0.05). This established that treatment with naringin was able to reduce insulin resistance. The OGTTs were performed at the end of the experiments. The mean values of the rats’ blood glucose at 0, 30, 60, 90, and 120 min showed that the AUC of the HFD group was significantly increased when compared to the control group (*p* < 0.01, Figure 1E). Conversely, the AUC value of both naringin concentrations of 50 and 100 mg/kg were significantly decreased when compared with the HFD group (*p* < 0.01, Figure 1E).

### 3.6. Effect of Naringin on Glucose Uptake, Protein Expressions, and Glycogen Content of Skeletal Muscles in High-Fat-Diet-Fed Rats

**Figure 2:**
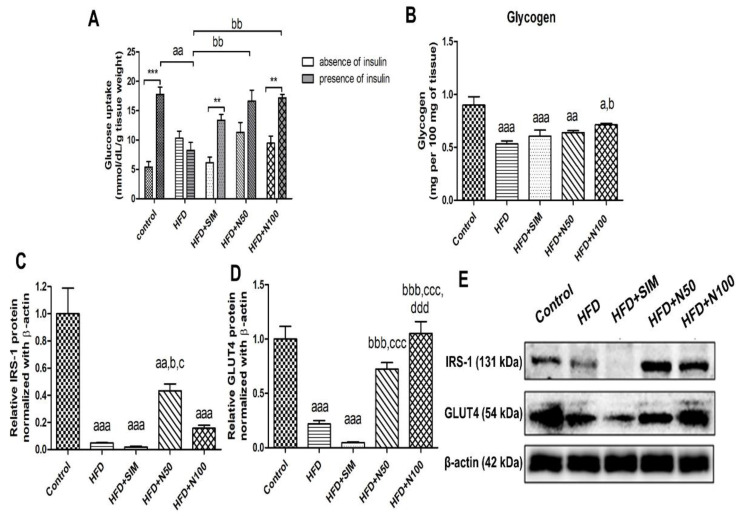
The effect of naringin on glucose uptake, glycogen content, and protein expressions in the quadriceps of the high-fat diet fed rats. Glucose uptake presence or absence insulin (**A**) and the glycogen content (**B**) in the quadriceps muscle tissues of the rats. (**E**) Representative protein expression images of IRS-1 (**C**) and GLUT4 (**D**) in the quadriceps tissues. Results are shown as mean ± SEM for 5 to 6 rats per group. **, *** *p* < 0.01 and 0.001 when compared to the absence of insulin. ^a^ *p* < 0.05, ^aa^ *p* < 0.01, and ^aaa^ *p* < 0.001 vs. the control group; ^b^ *p* < 0.05, ^bb^ *p* < 0.01, and ^bbb^ *p* < 0.001 vs. the HFD group; ^c^ *p* < 0.05, and ^ccc^ *p* < 0.001 vs. the HFD + SIM group; ^ddd^ *p* < 0.001 vs. the HFD group.

The glucose uptake by the skeletal muscles was determined by either the presence or absence of insulin (Figure 2A). In the presence of insulin, the control, simvastatin, and naringin groups significantly increased their levels of glucose uptake when compared with the absence of insulin within the same groups. The glucose uptake of the HFD group with presence of insulin was significantly lower than the control group (*p* < 0.01, Figure 2A), which led to the glycogen levels in the skeletal muscles being significantly lower than the control group. This was consistent with the expressions of both the IRS-1 and GLUT4 in the HFD group, which were significantly reduced when compared to the control group (*p* < 0.001, Figure 2C,D). Interestingly, the naringin-treated groups had significantly increased levels of glucose uptake in the skeletal muscles when compared to the HFD group’s presence of insulin (*p* < 0.01, Figure 2A), whereas there were no statistical differences when compared with the HFD + SIM group. The group treated with 100 mg/kg of naringin had high glycogen levels, and they showed statistical differences when compared with the HFD group (*p* < 0.05, Figure 2B). The expression of the GLUT4 was significantly increased when treated with 100 mg/kg of naringin (*p* < 0.001, Figure 2D), while the group treated with 50 mg/kg had significantly increased the levels of IRS1 (*p* < 0.05, Figure 2C) when compared to the HFD and the HFD + SIM groups.

### 3.7. Effect of Naringin on the Expression of Antioxidant Enzymes, Protein Metabolism mRNA in Quadriceps Muscle Tissues

**Figure 3:**
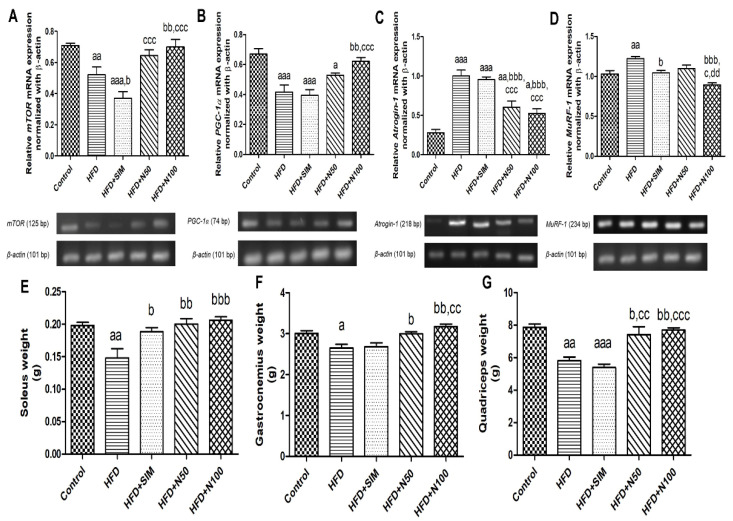
The effect of naringin on the mRNA expression of protein metabolism in the quadriceps of high-fat-diet-fed rats. Representative mRNA expression images of the quadriceps tissues. *mTOR* (**A**), *PGC-1α* (**B**), *Atrogin-1* (**C**), *MuRF-1* (**D**). The results are shown as mean ± SE from a duplicate of at least 6 rats. The figures of the muscle weights of the soleus, gastrocnemius, and quadriceps tissues of the rats are shown as (**E**–**G**), respectively. The results are shown as mean ± SEM for 6 to 7 rats per group as: ^a^ *p* < 0.05, ^aa^ *p* < 0.01, and ^aaa^ *p* < 0.001 vs. the control group; ^b^ *p* < 0.05, ^bb^ *p* < 0.01, and ^bbb^ *p* < 0.001 vs. the HFD group; ^c^ *p* < 0.05, ^cc^ *p* < 0.01, and ^ccc^ *p* < 0.001 vs. the HFD + SIM group; and ^dd^ *p* < 0.01 vs. the HFD + N50 group.

We analysed the gene expression of protein degradation (*Atrogin-1* and *MuRF-1*) and protein synthesis (*mTOR* and *PGC-1α*) in quadriceps tissues (Figure 3). The HFD and HFD + SIM groups showed that the mRNA expressions of the *mTOR* (*p* < 0.01 and *p* < 0.001, respectively, Figure 3A) and *PGC-1α* (*p* < 0.001, Figure 3B) were significantly reduced, whereas both *Atrogin-1* and *MuRF-1* genes in the HFD group were significantly increased (*p* < 0.001 and *p* < 0.01, respectively, Figure 3C,D) when compared to the control group. The *Atrogin-1* mRNA in HFD + SIM group was also significantly increased (*p* < 0.001) but had no statistical difference in *MuRF-1* mRNA when compared to the control group. On the other hand, the 100 mg/kg of naringin treated had significantly increased the mRNA expression of the *mTOR* and *PGC-1α* (*p* < 0.01, Figure 3A,B), while it showed significantly decreased expressions of protein degradation (*p* < 0.001, Figure 3C,D) when compared to the HFD groups. Therefore, the 100 mg/kg of naringin treatment was able to decrease the mRNA expression related to protein degradation, which may be one of the reasons that lead to the increase in the muscle mass of soleus, gastrocnemius, and quadriceps (*p* < 0.01, Figure 3E–G) when compared with the HFD group.

### 3.8. Effect of Naringin on Antioxidants and Lipid Peroxidation in High-Fat-Diet-Fed Rats

**Figure 4:**
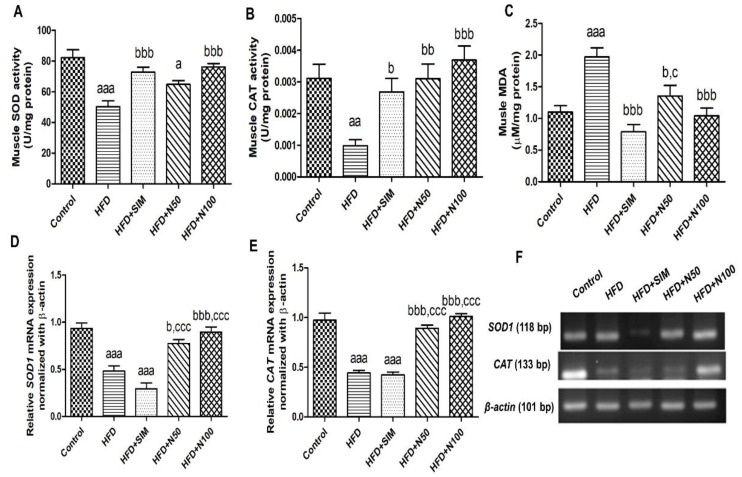
The effect of naringin on antioxidant activity and lipid peroxidation in high-fat-diet-fed rats. SOD (**A**), CAT (**B**), and MDA (**C**) of muscle tissue in rats. The mRNA expression of *SOD1* (**D**) and *CAT* (**E**) in the quadriceps muscle tissues of the rats. (**F**) Representative mRNA expression images of antioxidant enzymes in the quadriceps tissues. Results are shown as mean ± SEM for 6 rats per group. ^a^ *p* < 0.05, ^aa^ *p* < 0.01, and ^aaa^ *p* < 0.001 vs. the control group; ^b^ *p* < 0.05, ^bb^ *p* < 0.01, and ^bbb^ *p* < 0.001 vs. the HFD group; ^c^ *p* < 0.05, and ^ccc^ *p* < 0.001 vs. the HFD + SIM group.

The HFD group had significantly decreased levels of SOD and CAT activities (*p* < 0.001 and *p* < 0.01, respectively) while having notably increased levels of MDA in the skeletal muscle tissues (*p* < 0.001) when compared with the control group (Figure 4A–C). This is consistent with the expressions of *SOD1* and *CAT*, which were significantly lower than the control group (*p* < 0.001, Figure 4D,E). Conversely, the groups treated 100 mg/kg of naringin had significantly increased levels of SOD and CAT in the muscle tissues (*p* < 0.001, Figure 4A,B) when compared with the HFD group. The levels of muscle MDA in both the 100 mg/kg of naringin- and simvastatin-treated groups were significantly reduced (*p* < 0.001, Figure 4C) when compared with the HFD group (Figure 4C). In addition, the 100 mg/kg of naringin group demonstrated increased mRNA expressions of both *SOD1* and *CAT* when compared with the HFD and HFD + SIM groups (*p* < 0.001, Figure 4D–F).

### 3.9. Histopathology of Quadriceps Muscles in High-FatDiet-Fed Rats

**Figure 5:**
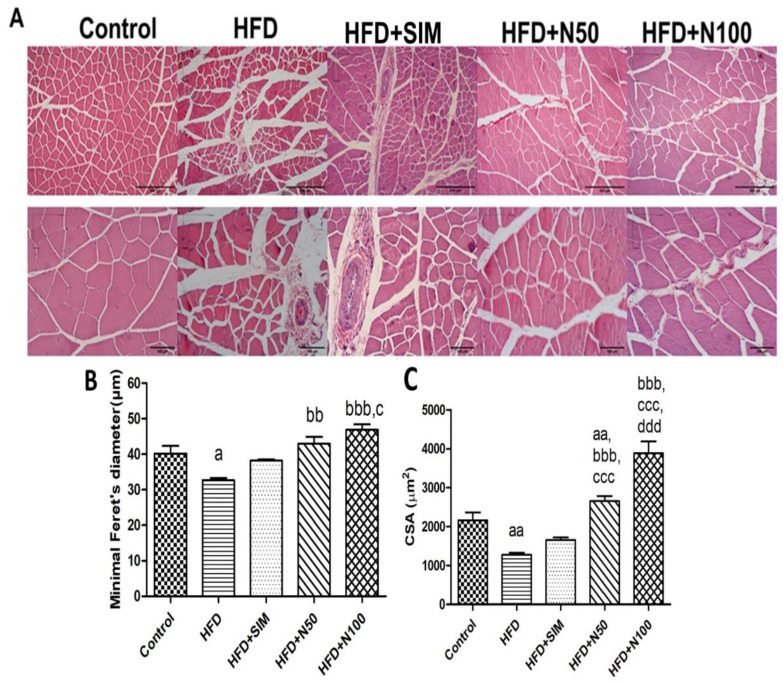
The effect of naringin on the morphology of high-fat-diet-fed rats. Representative H&E-stained images of the quadriceps were shown at 40X (scale bar = 200 μm) and 100X magnification (scale bar = 100 μm) (**A**). Minimal Feret’s diameters (fibre diameter) (**B**) and cross-section area (fibre area) (**C**) were calculated. Results are shown as mean ± SEM for 5 to 7 rats per group. ^a^ *p* < 0.05, and ^aa^ *p* < 0.01 vs. the control group; ^bb^ *p* < 0.01, and ^bbb^ *p* < 0.001 vs. the HFD group; ^c^ *p* < 0.05, and ^ccc^ *p* < 0.001 vs. the HFD + SIM group; and ^ddd^ *p* < 0.01 vs. the HFD + N50 group.

The control group showed normal morphology of quadriceps muscles (Figure 5A), whereas the HFD groups showed that the fibre diameter and cross-section area (CSA) were lower than the control group (*p* < 0.05 and *p* < 0.01, respectively, Figure 5B,C). However, the groups treated with 50 and 100 mg/kg of naringin had significantly increased diameter (*p* < 0.01 and *p* < 0.001, respectively, Figure 5B) and CSA (*p* < 0.001, Figure 5C) of fibre when compared with the HFD group. Interestingly, the 100 mg/kg of naringin group showed that the fibre diameter (*p* < 0.05) and CSA (*p* < 0.001) was significantly higher than the HFD + SIM (Figure 5B,C).

## 4. Discussion

The consumption of a high-fat diet causes an increased accumulation of lipids in the plasma and various tissues, which induces obesity. The HFD group had significantly increased levels of TC and TG in their muscles when compared with the rats fed a normal diet, whereas the naringin-treated groups effectively reduced the levels of TG and TC in the rat’s muscle tissues. Moderate reduced caloric intake by 20–40% demonstrated changes in body compositions and metabolic indicators, including lipids [28,29]. On the contrary, we found slightly decreased calories (<7%) in the naringin and simvastatin treated groups, compared to the HFD, while alterations in BW and other metabolic parameters were notably observed after the treatment. Therefore, the regulation of caloric intake is unlikely to be the mechanism contributing to the metabolic alterations in the present study. Several studies reported that a 100 mg/kg of naringin supplement, reduced both the TC and TG in plasma of the rats that were induced with a HFD [14,30], which may be due to the inhibitory effects of naringin on the digestive enzymes. The in vitro part of this study showed that naringin can inhibit both the pancreatic lipase and pancreatic cholesterol esterase. These are the two important digestive enzymes for the hydrolysis of fats, including triglyceride and cholesterol ester. If the enzymes are inhibited, the digestion of fat can be reduced, which prevents its absorption and accumulation. Similarly, the study by Makynen [18] found that naringin in six pomelo cultivars reacted against pancreatic lipase and pancreatic cholesterol esterase. Naringin not only had the ability to inhibit the pancreatic lipase and pancreatic cholesterol esterase but also α-glucosidase. This is consistent with a report by Priscilla [31]. Under normal conditions, α-glucosidase plays the role of digestive enzymes that degrades glucose by changing polysaccharides into monosaccharides, which are then absorbed into to the intestinal lumen and transported by blood circulation. The inhibitory abilities of naringin to the digestive enzymes, results in the decreased absorption of glucose and lipids into the circulation. Therefore, naringin has anti-glycaemic and anti-dyslipidaemic activities [12]. The effect against hyperlipidaemia is similar to anti-dyslipidaemic drugs such as statins that can reduce lipids, but these drugs have been reported to show side effects on the skeletal muscles [7,8]. 

An over-accumulation of lipids in skeletal muscle tissues affects the glucose, protein, and lipid metabolisms by reducing GLUT4 translocation into muscles and the muscle protein imbalance via the IRS-1/Akt/mTOR pathway. In addition, obesity also affects the impairment of the regeneration and function of skeletal muscles, including force of muscle contraction and the growth of the muscle fibres, which leads to muscle atrophy [1,32]. The force of muscle contraction depends on the number and size of the fibres, which can be determined from the muscle fibres’ morphology [33]. In this study, we found that the rats that received the high-fat diet were obese by showing a body weight gain of approximately 24% when compared to the control group. This led to the accumulation of TG and TC in the muscle tissues and consequently caused abnormalities in the muscle morphology. Obesity is associated with muscle atrophy by the attenuated growth of muscle fibres. It is also characterized by morphological changes, which include the number, size, and CSA of reduced fibres, leading to an impaired force of muscle contraction and loss of muscle fibres [33,34,35]. However, muscle contractions depend on the number and size of the fibres [33]. The analysis of the muscle fibres was calculated by measuring the CSA, and the diameter [27]. These results showed that the HFD group had a decreased diameter, and CSA in their skeletal muscles, which led to muscle fibre atrophy, when compared to the control group. Previous studies showed that the high-fat-diet-fed rats CSA, number of fibres, and diameters of their soleus and quadriceps had decreased, which led to reduced muscle weight and also impaired force of muscle contraction [27,34,35]. Conversely, the skeletal muscle parameters from the histological analysis in our study of the naringin treatment were higher than both the HFD and the HFD + SIM groups. A previous study showed that the effect of statin on the morphology of muscle fibres, induced myopathy, which comprised muscle atrophy, necrosis, and inflammation [36]. Similarly, the histological aspects of the muscle fibres in the HFD + SIM group in our study were split and had irregular shapes and sizes when compared with the control and naringin treatment groups, which is consistent with the previous study [9]. The naringin supplements were beneficial in improving the regeneration of the muscle fibres and had no side effects when compared to the statins used to treat hyperlipidaemia.

Excess intake of a high-fat diet induces obesity, and the common characteristics are accumulated FFAs that lead to an increase in their oxidation in the mitochondria, which in turn induces ROS production that affects oxidative stress [6]. This is caused by an imbalance of increased ROS production and decreased antioxidant enzymes. In addition, MDA is a product of lipid peroxidation that is a marker for oxidative stress. This study found that the HFD group had decreased SOD and CAT activities while, at the same time, had increased MDA level in the skeletal muscle tissues when compared with the control group. Naringin is a flavonoid found in citrus fruits, which has been reported to not only have lipid-lowering activity but also exhibits anti-oxidative capability against oxidative stress and contains natural antioxidant properties [14]. This study showed that naringin has strong antioxidant activities for scavenging free radicals, which were determined by ABTS, DPPH radical scavenging assay, and total phenolic content, which is consistent with a report by Vallejo [37]. The results showed that the rats that received 50 and 100 mg/kg of naringin had increased levels of SOD and CAT activities in the skeletal muscle tissues when compared with the HFD group. Additionally, we found that antioxidant gene expressions, including *SOD1* and *CAT*, increased in the skeletal muscles of the naringin-treated groups. The study by Attia [38] found that 5–50 mg/kg of naringin had enzymatic antioxidants. Similarly, naringin treatment significantly decreased MDA levels and increased enzymatic antioxidants, which is consistent with a report by Attia [38]. Therefore, naringin has properties against oxidative stress due to strong superoxide and H_2_O_2_ scavenging activities [13].

Obesity-related increase in FFA concentrations induces insulin resistance by increasing ROS products. This attenuates the mechanism of the IRS-1/Akt/mTOR signalling pathway, which leads to an imbalance between protein synthesis and breakdown within the muscle that causes loss of muscle mass or atrophy [39,40]. Despite the fact that our result did not determine the expression of phosphorylation of the IRS-1 and GLUT4, the decreased protein expressions of IRS-1 can demonstrate the insulin resistance in skeletal muscle. These contribute to impaired glucose metabolism and decreased muscle mass, which are the important pathological features of muscle atrophy in insulin resistance in HFD-induced obesity [41]. In fact, several studies used the protein expressions of IRS-1 and GLUT4 to determine the insulin signalling related to insulin resistance [42,43,44]. This is due to IRS-1 being an essential element for promoting insulin-induced glucose uptake by stimulating GLUT4 in the muscle. Our results demonstrated, in accordance with other studies, that the protein expressions of IRS-1, not the phospho-IRS-1 and GLUT4, were significantly reduced in the muscle of animals fed a high-fat diet when compared with the control group [43,45]. In addition, insulin resistance is associated with hyperinsulinemia and hyperglycaemia due to impaired insulin signalling of those effects, increasing the amount of insulin and glucose in the circulation system and reducing the levels of glucose uptake and glycogen. This led to reduced energy storage in muscles since glycogen is important as an energy source in the anaerobic glycolysis process for the contraction of muscles [3,23]. In this study, the levels of glucose uptake and glycogen content in the HFD and simvastatin groups were lower than both the control and the naringin treatment groups, which were consistent with the results of the expressions of IRS-1 and GLUT4 proteins being reduced in the skeletal muscles. The high value of HOMA-IR, blood glucose, serum insulin, and AUC in the HFD group demonstrated that insulin resistance and/or decreased insulin sensitivity occurred. A previous study reported that insulin resistance was increased in the high-fat-diet-induced obese rats, which led to abnormal muscle metabolism [32]. In addition, several studies found that simvastatin induced insulin resistance by suppressing Akt/mTOR signalling pathway, which affects the GLUT4 translocation into the membrane, which may then reduce glucose uptake into the muscle cells (L6 and C_2_C_12_) [46,47]. This causes an increase in glucose in the blood, which leads to insulin resistance. Interestingly, the naringin groups increased insulin sensitivity by increasing glucose uptake, glycogen levels, and the expressions of GLUT4 and IRS-1 proteins in the skeletal muscles, which, at the same time, decreases blood glucose levels and serum insulin when compared to the HFD group [15]. Our result is in accordance with the report by Kumar [48], who suggested that 100 mg/kg/d of naringin significantly decreased blood glucose and serum insulin in high-fat-diet-fed rats. In addition, the supplementation of naringin that reduced insulin resistance was measured by the value of the HOMA index and was shown to be lower than the HFD-fed rats [49]. Therefore, naringin is able to treat obesity-related insulin resistance that leads to type 2 diabetes mellitus and can also control glucose homeostasis. The present study indicated that naringin stimulates the glucose entry into the muscle cells via the GLUT4 carrier and is stored in the form of glycogen for energy, and at the same time, the blood glucose is reduced. Therefore, naringin is also able to attenuate hyperglycaemia and hyperinsulinemia that leads to reduced insulin resistance in obesity.

In addition, insulin plays an important part in controlling protein synthesis and degradation via the IRS-1/Akt/mTOR signalling pathways. Akt activation is induced by IGF1 and/or insulin that activates the mTOR pathway, which controls protein synthesis while inhibiting the FOXO pathway. In addition, insulin signalling could stimulate protein synthesis in muscle tissue via activating the expression of the *mTOR* gene. Shin reported that the expression of *mTOR* mRNA in the HF group was lower than the control group [50]. In addition, the mTOR is associated with the regulator of muscle hypertrophy. The upregulation of *mTOR* gene expression was reported in skeletal muscle of older women with resistance exercise training combined with PUFA comparing to the untrained subjects [51]. Therefore, we used the expression of *mTOR* mRNA to indicate protein synthesis that is associated with insulin signalling. The increased expression of *PGC1α*, which regulates mitochondrial biogenesis and takes part in the protective role against skeletal muscle atrophy, was observed in the naringin-treated group when compared with the HFD and HFD + SIM groups. *Atrogin-1* and *MuRF-1* promotes protein degradation in the muscle cells, while, at the same time, ROS products are able to activate the FOXO pathway, which leads to reduced muscle mass due to increased expression of protein degradation [2,52]. Several studies have reported that obesity negatively alters the protein turnover rate in the skeletal muscles, which is associated with the imbalance between protein synthesis and protein degradation [3,39]. The results showed that the HFD and simvastatin groups had significantly increased mRNA expressions of both *Atrogin-1* and *MuRF-1* which are the genes related to skeletal muscle atrophy [53], while having reduced expressions of *SOD1*, *CAT*, *mTOR*, and *PGC-1α* mRNA in the skeletal muscle tissue when compared with the control group. This led to the decreased weight and proteins of muscles in obese rats, which is consistent with the previous study [54]. Hasan [55] reported that increased expressions of *FOXO3*, *Atrogin-1/MAFbx*, and *MuRF-1* and a decreased expression of *PGC-1α*, which plays a role as a mitochondrial biogenesis regulator in the skeletal muscle of the HFD-induced obesity rats, was attributed to insulin resistance of the muscles, which was consistent with a report by Abrigo et al. [56] and our study. As previously reported, the expressions of *Atrogin-1* and *MuRF-1* mRNA were increased after treatment with simvastatin, while the expression of *PGC-1α* mRNA was decreased which led to muscle atrophy due to the process of protein degradation being higher than the protein synthesis [7,57]. Interestingly, we demonstrated that naringin can reduced insulin resistance and increase the mRNA expressions of *SOD1*, *CAT*, *mTOR*, and *PGC-1α*, while showing significantly decreased expressions of *Atrogin-1* and *MuRF-1* mRNA. Our results exhibited evidence to show that naringin supplementation is able to increase muscle mass by increasing the expression of *mTOR* while decreasing the protein breakdown expression in the muscle cells. This result is in company with the increased muscle weight found in quadriceps, soleus, and gastrocnemius muscles and the improvement in muscle abnormalities in the histological study. Therefore, naringin is able to maintain the rate of protein metabolism while increasing the protein content and mass in the skeletal muscles.

ALT, AST, CK, and LDH are commonly used to evaluate tissue injuries, including liver and skeletal muscle injuries [58]. Several studies have shown that the serum AST, ALT, CK, and LDH levels were significantly increased in the high-fat-diet-induced obese rats when compared to the control group [59,60]. These results suggested that the HFD induced the impairment of both the liver and skeletal muscle functions, which caused dyslipidaemia and insulin resistance [61]. In this study, the serum levels of AST, ALT, CK, and LDH in the HFD group were higher than the control group, whereas they were reduced in the naringin treatment groups when compared to the HFD and simvastatin groups. Alam [14] reported that 100 mg/kg of naringin reduced plasma AST and ALT in high-carbohydrate, high-fat-diet-fed rats. In addition, 10 mg/kg body weight of naringin significantly decreased serum concentrations of AST, ALT, CK, and LDH in doxorubicin-induced cardiotoxicity in mice [62]. The simvastatin group had increased the levels of AST, CK, and LDH in the serum when compared to the control groups, which is consistent with previous research [63,64]. Furthermore, statins have been shown to impact the insulin signalling pathway and increase the risk of new-onset diabetes. This study showed that simvastatin is an effective drug for treating dyslipidaemia and obesity, but it effects the skeletal muscles. However, naringin had no side-effects on the muscles and demonstrated a reduction in CK, LDH, and AST that led to improving the performance of muscles in the high-fat-diet-induced obese rats.

Naringin treatment has no side effects and improves skeletal muscle regeneration by improving insulin resistance and reducing oxidative stress and the expressions of both *Atrogin-1* and *MuRF-1*, which results in decreased protein degradation in high-fat-diet-induced obese rats. In addition, naringin was also able to increase expressions of GLUT4, IRS-1, *mTOR*, and *PGC-1α* levels that demonstrated improved weight of muscles. Therefore, the naringin compound can be used as an alternative approach for the treatment of hyperlipidaemia and hyperglycaemia, which affects the muscles. Scalbert [65] stated that the recommended intake of naringin for humans is 1 g/d. The doses of 50 and 100 mg/kg of naringin were used in this study and reported not to be toxic to laboratory animals. The daily human intake of naringin can be calculated using the human equivalent dose (HED) formula by Nair [66]. The estimated daily intake of naringin is 486.60 and 973.20 mg/d for humans that weigh 60 kg when compared with the rats at doses of 50 and 100 mg/kg, respectively. Therefore, the doses that we used in this study were safe for daily human consumption.

## 5. Conclusions

**Figure 6:**
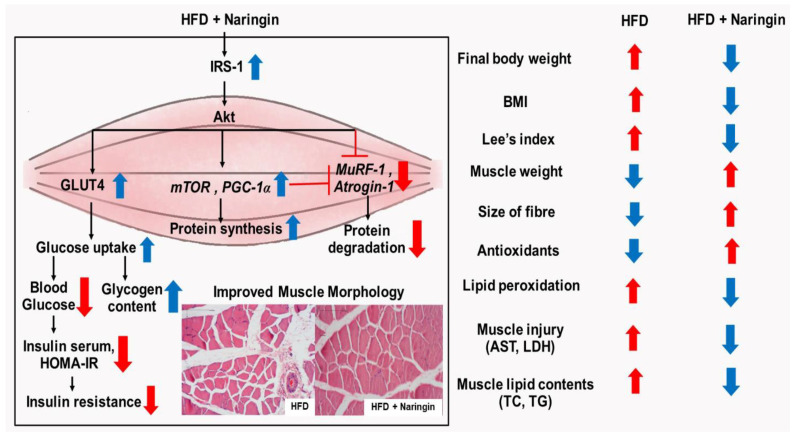
Schematic diagram of the possible underlying mechanism of naringin to regulate protein turnover in high-fat diet-induced obese rats. Naringin reduced the levels of muscle lipid accumulation, which causes oxidative stress, by increasing antioxidant enzymes and not only decreasing the malondialdehyde (MDA) production of lipid peroxidation but also the lactate dehydrogenase (LDH) and aspartate aminotransferase (AST), which are the indicators of muscle injury. Naringin increased the mRNA expressions of *mTOR* and *PGC-1α* and also the protein expressions of IRS-1 and GLUT4. In addition, it increased levels of glucose uptake and glycogen content while inhibiting the mRNA expression of *Atrogin-1* and *MuRF-1*. This leads to increased muscle mass and size of the fibres, improvement in insulin sensitivity, and a decline in insulin resistance of skeletal muscles in high-fat-diet- induced obese rats.

In summary, this study showed that naringin and simvastatin both have the potential to reduce body weight, muscle lipid accumulation, and MDA levels while increasing antioxidant enzyme activities in both the serum and the quadriceps. Naringin not only had improved insulin resistance and increased the gene expression of the antioxidant enzymes (*SOD1* and *CAT*), *mTOR*, and *PGC-1α*, but the expressions of IRS-1 and GLUT4 proteins were also increased, which led to increases in the glucose uptake and the glycogen levels. At the same time, oxidative stress decreased, leading to reduced expression of both *MuRF1* and *Atrogin-1* genes in the quadriceps, which play an essential role in protein degradation. This study demonstrates that naringin supplementation increases muscle weight by reducing the expression of protein degradation and increasing the expression of protein synthesis and mitochondrial biogenesis, whereas the simvastatin treatment increased the mRNA expression of *Atrogin-1*, which led to the loss of muscle mass. Interestingly, a side effect of simvastatin caused myotoxicity in the muscles, which showed that the levels of muscle injury indices were higher than the naringin treatment and effectively reduced the levels of glucose uptake and glycogen in the muscles. Naringin also improved the morphological change of the muscle fibres and showed increased muscle area and diameter. Taken together, the results of our experiments show that naringin, which is a compound that occurs naturally in citrus fruits, has no side-effects on the muscles, therefore, it is an alternative supplement that benefits the treatment of dyslipidaemia, obesity-induced muscle dysfunction, and at the same time decreases insulin resistance and restores sensitivity in high-fat-diet-induced obese rats (Figure 6).

## Figures and Tables

**Table 1:** Primer sequences for polymerase chain reaction.

Gene		Primer Sequence (5′-3′)	Amplification Size (bp)	Annealing Tm (°C)
***SOD1***	F:	AATGTGTCCATTGAAGATCGTGTG	118	60
	R:	GCTTCCAGCATTTCCAGTCTTTGTA		
***CAT***	F:	GCAGGAAGACTTGCACACGGA	133	58
	R:	ATGGGAAGGTTTCTGCCTCC		
***MuRF-1***	F:	GCCATCCTGGACGAGAAGAA	234	55
	R:	CAGCTGGCAGCCCTTGGA		
***Atrogin-1***	F:	AGACCGGCTACTGTGGAAGAG	218	60
	R:	CCGTGCATGGATGGTCAGTG		
***mTOR***	F:	TCCACTGGAAGCACAGACCAAG	125	58
	R:	GCTTATCAAGCAAGCGACATCTCA		
***PGC-1α***	F:	GCGCCGTGTGATTTACGT	74	56
	R:	AAAACTTCAAAGCGGTCTCTCAA		
***β-actin***	F:	TGTCCACCTTCCAGCAGATGT	101	58
	R:	AGCTCAGTAACAGTCGCGCTAGA		

Superoxide dismutase 1 (*SOD1*), Catalase (*CAT*), Muscle RING-finger protein-1 (*MuRF1*), Muscle atrophy F-box (*Atrogin-1*), mammalian target of rapamycin (*mTOR*), Peroxisome proliferator-activated receptor co-activator-1α (*PGC-1α*), Beta-actin (*β-actin*), Forward (F), and Reverse (R).

**Table 2:** IC50 values and % inhibition of antioxidant activity assays and enzymes in the digestive system of naringin.

Assay	Naringin	Positive Control When Compared with Sample		
		Acarbose	Simvastatin	Orlistat
TPC (mgGAE/gDW)	1.72	N/A	N/A	N/A
DPPH (molAAE/gDW)	3.30	N/A	N/A	N/A
IC50 values of DPPH (mg/mL)	24.82	N/A	N/A	N/A
ABTS (molTE/gDW)	0.17	N/A	N/A	N/A
IC50 values of ABTS (mg/mL)	2.67	N/A	N/A	N/A
α-glucosidase				
IC50 value (mg/mL)	0.259	1.025	N/A	N/A
Maximum of inhibition (%)	81.22 ± 0.35	88.27 ± 0.13	N/A	N/A
Cholesterol esterase				
IC50 value (mg/mL)	0.21	N/A	0.92	N/A
Maximum of inhibition (%)	97.26 ± 0.88	N/A	82.40 ± 0.38	N/A
Pancreatic lipase				
IC50 value (mg/mL)	0.009	N/A	N/A	0.0002
Maximum of inhibition (%)	71.21 ± 3.29	N/A	N/A	84.99 ± 1.05

IC50 was inhibitory concentration at 50% of naringin. Results are shown as mean ± SE from a triplicate of experiments. N/A: not applicable, mgGAE/gDW: milligram gallic acid equivalent per gram dry weight of sample, molAAE/gDW: mole ascorbic acid equivalent per gram dry weight of sample, molTE/gDW: mole Trolox equivalent per gram dry weight of sample.

**Table 3:** The effects of naringin on food intake, body weight, Lee’s index, BMI, %adiposity index, and biochemistry parameter levels in high-fat-diet-fed rats.

Parameters	Control	HFD	HFD + SIM	HFD + N50	HFD + N100
**Initial weight (g)**	255.32 ± 19.57	257.79 ± 22.04	245.06 ± 30.69	249.62 ± 15.98	237.80 ± 14.66
**Final weight (g)**	516.20 ± 15.61	601.40 ± 2.71 ^aaa^	555.20 ± 7.79 ^a,bb^	568.60 ± 5.27 ^aa^	556.20 ± 7.83 ^a,b^
**Lee’s index (g/cm)**	308.38 ± 2.71	321.14 ± 2.5 ^aaa^	316.19 ± 3.49	313.54 ± 2.18	312.68 ± 0.89 ^b^
**BMI (kg/m^2^)**	0.69 ± 0.03	0.79 ± 0.03 ^aaa^	0.75 ± 0.03 ^aa,b^	0.74 ± 0.03 ^a,b^	0.74 ± 0.02 ^a,bb^
**Caloric intake (kacl/day)**	79.78 ± 1.41	90.78 ± 3.27 ^aa^	84.39 ± 2.19	84.04 ± 2.26	83.57 ± 2.50
**Muscle TC (mg/g of tissue)**	134.42 ± 10.63	218.76 ± 23.01 ^a^	120.07 ± 8.74 ^bb^	91.94 ± 7.56 ^bbb^	69.49 ± 12.74 ^bbb^
**Muscle TG (mg/g of tissue)**	410.28 ± 29.14	1217.18 ± 53.13 ^aaa^	418.40 ± 35.71 ^bbb^	877.05 ± 38.03 ^aaa,bbb,ccc^	626.39 ± 28.94 ^aaa,bbb,ccc,ddd^
**Serum CK (units/L)**	42.67 ± 2.68	56.08 ± 6.32	85.24 ± 12.19 ^a^	49.01 ± 4.74 ^c^	46.87 ± 6.94 ^c^
**Serum LDH (nmol)**	101.84 ± 15.50	147.65 ± 9.97	175.16 ± 43.07	118.9 ± 11.78	88.98 ± 20.31 ^b^
**Serum AST (U/L)**	92.4 ± 3.59	223.4 ± 21.08 ^aaa^	191.8 ± 14.65 ^aaa^	174.2 ± 21.24 ^aa,b^	173 ± 13.33 ^aa,b^
**Serum ALT (U/L)**	24.25 ± 1.25	89.5 ± 15.50 ^aa^	75 ± 15.07 ^aa^	58 ± 10.46	59.75 ± 11.34

Results are shown as mean ± SEM for 5 to 6 rats per group. BMI: Body mass index, TC: Total cholesterol, TG: Triglycerol, CK: Creatine Kinase, LDH: Lactate dehydrogenase, AST: Aspartate transaminase, ALT: Alanine transaminase. ^a^ *p* < 0.05, ^aa^ *p* < 0.01, and ^aaa^ *p* < 0.001 vs. the control group; ^b^ *p* < 0.05, ^bb^ *p* < 0.01, and ^bbb^ *p* < 0.001 vs. the HFD group; ^c^ *p* < 0.05, and ^ccc^ *p* < 0.001 vs. the HFD + SIM group; and ^ddd^ *p* < 0.001 vs. the HFD + N50 group.

## Data Availability

The data presented in this study are available from the corresponding author on reasonable request.

## Abbreviations

ABTS	2,2′-azino-bis (3′-ethylbenzthiazoline-6-sulphonic acid)
*Atrogin-1/MAFbx*	muscle-specific F-box
*CAT*	catalase
CSA	cross-sectional area
DPPH	2,2-diphenyl-1-picrylhydrazyl
FBG	Fasting blood glucose
FOXO	Forkhead Box O
GLUT4	Glucose transporter type 4
HFD	high-fat diet
IRS-1	insulin receptor substrate 1
MDA	malondialdehyde
*mTOR*	mechanistic target of rapamycin
*MuRF-1*	muscle ring-finger protein-1
OGTT	Oral glucose tolerance test
*PGC-1α*	peroxisome proliferator-activated receptor gamma coactivator 1-alpha
ROS	reactive oxygen species
SOD	superoxide dismutase
TC	total cholesterol
TG	triglyceride
TPC	total phenolic content
